# Exploring the role of R&D collaborations and non-patent IP policies in government technology transfer performance: Evidence from U.S. federal agencies (1999–2016)

**DOI:** 10.1371/journal.pone.0268828

**Published:** 2022-05-24

**Authors:** Iman Hemmatian, Todd A. Ponzio, Amol M. Joshi

**Affiliations:** 1 College of Business Administration, Cal Poly Pomona, Pomona, California, United States of America; 2 School of Medicine, Wake Forest University, Winston-Salem, North Carolina, United States of America; 3 School of Business, Wake Forest University, Winston-Salem, North Carolina, United States of America; German University in Cairo, CZECH REPUBLIC

## Abstract

Around the world, governments make substantial investments in public sector research and development (R&D) entities and activities to generate major scientific and technical advances that may catalyze long-term economic growth. Institutions ranging from the Chinese Academy of Sciences to the French National Centre for Scientific Research to the Helmholtz Association of German Research Centers conduct basic and applied R&D to create commercially valuable knowledge that supports the innovation goals of their respective government sponsors. Globally, the single largest public sector R&D sponsor is the U.S. federal government. In 2019 alone, the U.S. government allocated over $14.9 billion to federally funded research and development centers (FFRDCs), also known as national labs. However, little is known about how federal agencies’ utilization of FFRDCs, their modes of R&D collaboration, and their adoption of non-patent intellectual property (IP) policies (copyright protection and materials transfer agreements) affect agency-level performance in technology transfer. In particular, the lack of standardized metrics for quantitatively evaluating government entities’ effectiveness in managing innovation is a critical unresolved issue. We address this issue by conducting exploratory empirical analyses of federal agencies’ innovation management activities using both supply-side (filing ratio, transfer rate, and licensing success rate) and demand-side (licensing income and portfolio exclusivity) outcome metrics. We find economically significant effects of external R&D collaborations and non-patent IP policies on the technology transfer performance of 10 major federal executive branch agencies (fiscal years 1999–2016). We discuss the scholarly, managerial, and policy implications for ongoing and future evaluations of technology transfer at federal labs. We offer new insights and guidance on how critical differences in federal agencies’ interpretation and implementation of their R&D management practices in pursuit of their respective missions affect their technology transfer performance outcomes. We generalize key findings to address the broader innovation processes of public sector R&D entities worldwide.

## Introduction

As highlighted in a recent study by the World Intellectual Property Organization (WIPO), governments in many countries significantly expanded and accelerated their investments in research and development (R&D) activities as part of their policy responses to the 2009 financial crisis and the 2020 coronavirus outbreak [1]. For example, China is focused on building critical nationwide infrastructure via the construction of advanced data centers, 5G wireless networks, and new energy vehicles [1, 2]. These initiatives are driven primarily by greater public sector investment in the Chinese Academy of Sciences, which in 2018 announced plans to rapidly grow its number of national labs from 200 to 700 by 2020, with a fully operational key lab system expected to be completed by 2025. In another example, specifically for combating the coronavirus, France pledged 5 billion euros in R&D spending, which represents a 25% increase over its original R&D budget for 2020. This effort is led by the 10 research institutes that make up the French National Centre for Scientific Research (CNRS) and receive 80% of all public R&D funds allocated by the French government [3, 4]. In a similar effort within Europe, Germany’s second stimulus package, which targets COVID-19 recovery, features 50 billion euros of R&D investments in a wide array of future-focused technologies. These funds are directed towards R&D projects conducted by three distinct networks of federal- and state-sponsored labs, which include the Helmholtz Association of German Research Centers, the Max Planck Institutes, and the Fraunhofer Institutes [5, 6]. In other countries such as Turkey [7], India [8], and Israel [9], governments initiated similar programs to promote technology commercialization and spark growth. Despite the considerable differences in political systems and economic priorities across China, France, Germany, Israel, Turkey, and India, what their respective public sector R&D entities all have in common are clearly defined government mandates to pursue scientific and technical breakthroughs that may fuel long-term growth, prosperity, and security.

In line with its counterparts in the aforementioned countries, but on an even broader scale, the single largest public sector R&D sponsor in the world is the U.S. federal government, which has a similar pro-growth mandate to drive scientific discovery, develop new knowledge, promote technical standards, and generate useful innovations. For instance, in 2019 alone, the U.S. government funded an estimated total of $141.5 billion in R&D expenditures [10], which “plays an irreplaceable role in directing technology toward more general and active domains” [11, 12]. Approximately 27% or $39.6 billion of this total is intramural R&D conducted internally by federal agencies, while the bulk of this funding, 73% or $101.9 billion, is allocated to R&D conducted externally by for-profit corporations and nonprofit organizations. Within the extramural R&D allocation, industry represents $43.6 billion, universities receive $33.4 billion, and contractor-operated federally funded research and development centers (FFRDCs, many of which are called ‘national labs’ or ‘federal labs’) account for $14.9 billion. Although there is an established stream of prior research on university-industry technology transfer [13–20], far less is known about technology transfer at national/federal labs and non-university research institutes. Despite governments’ consistently large and increasing budget allocations to public sector R&D entities and activities within their respective countries, there appears to be inconsistent and limited use of quantitative metrics for measuring performance outcomes related to creating and commercializing new technologies.

The dearth of research in this area is surprising because national labs are an essential component of the core systems of innovation in the U.S. and around the world [21, 22]. We aim to extend prior research in a new direction by exploring how government-industry technology transfer at national/federal facilities may differ from university-industry technology transfer along critical dimensions, especially in terms of the identification, adoption, and usage of appropriate performance metrics. Indeed, an evaluation of institutional policies and practices at these R&D facilities may yield new managerial and theoretical insights for improving the effectiveness of existing government-supported technology transfer processes. Our study investigates the following research question: *How do differences in R&D policy implementation across federal agencies affect their technology transfer activities and performance*? We believe that obtaining empirical evidence to answer this question is timely, relevant, and strategically important as governments around the world continue to expand the scale and scope of their funding for public sector R&D. The central premise of our study is that two main elements of federal agencies’ varying approaches to innovation management directly influence technology transfer performance: (1) their engagement in external R&D collaborations, through formal or informal partnership agreements; and (2) their adoption of policies for sharing non-patented intellectual property (IP). Although the importance of participating in external R&D collaborations and adopting policies for handling IP are routinely incorporated into existing research on university-industry transfer [23, 24], these factors are not yet systematically integrated into the emerging stream of research on government-industry technology transfer [25]. Our study aims to contribute to the nascent literature on federal technology transfer by providing a conceptual framework, expanded metrics to measure successful technology transfer, and fresh empirical evidence to guide scholars, managers, and policymakers in their evaluation of R&D commercialization processes.

Our study differs from previous studies in two critical ways. First, unlike prior research on government and academic technology transfer that focuses primarily on protecting proprietary technologies through *patenting* activities [26, 27], we examine the importance of external R&D *collaborations* and *sharing* proprietary technologies through *non-patent IP* policies such as copyrights and materials transfer agreements (MTAs). Second, in contrast to the emerging set of studies on federal technology transfer that use only agency-driven *supply-side* metrics to evaluate agency performance [28], we introduce customer-driven *demand-side* metrics and integrate both types of measures into our empirical analyses. For example, beyond the traditional supply-side metrics of *filing ratio*, *transfer rate*, and *licensing success rate* [29, 30] that capture a producer’s ability to *push* technologies into the commercial marketplace, we use the demand-side metrics of *licensing income* and *portfolio exclusivity* that capture a customer’s willingness to *pull* technologies out of government labs [31]. By incorporating external R&D collaborations and non-patent IP policies as *predictors* and demand-side metrics as *outcomes* in our models, we seek to provide a more holistic picture of federal technology transfer performance at the agency level.

We organize our study by first explaining the historical context of key legislative acts and proposing a conceptual framework. We then conduct a set of exploratory analyses that offer initial empirical evidence for the effects of external R&D collaborations and non-patent IP policies on the technology transfer performance of 10 major federal executive branch agencies (fiscal years 1999–2016). Overall, when we specifically examine agencies’ use of formal agreements for partnerships, we find a positive and significant relationship between this type of external R&D collaboration and all of our supply-side metrics (filing ratio, transfer rate, and licensing success rate), as well as portfolio exclusivity on the demand-side. In contrast, agencies’ use of other types of customized and informal external R&D collaborations appears to be associated with two main effects on the demand-side: (1) a significant *decrease* in licensing income; and (2) a simultaneous and somewhat surprising corresponding *increase* in portfolio exclusivity. We find evidence that agencies with a greater utilization of FFRDCs have lower agency-driven technology transfer performance in terms of supply-side metrics. On the demand-side, we find that greater FFRDC utilization is associated with greater licensing income and lower portfolio exclusivity.

We also find the adoption of non-patent IP policies to be associated with substantial and economically significant shifts in the supply-side *and* demand-side metrics. While the supply-side effects are similar for copyright agreements and MTAs, the demand-side effects are different. External R&D collaborations appear to further amplify these observed effects. In sum, our findings indicate that federal technology managers must carefully consider the combined effects of external R&D collaboration and non-patent IP policies when formulating their respective agencies’ technology transfer plans and programs. Based on these findings, we discuss the scholarly, managerial, and policy implications for ongoing and future evaluations of federal agencies’ technology transfer performance. Beyond U.S. federal agencies, we also consider how our key findings may inform possible innovation process improvements and policy reforms for public sector R&D entities and activities in other countries and contexts.

## Historical context and conceptual framework

### Historical context

The *Bayh-Dole Act of 1980* (Bayh-Dole), the *Stevenson-Wydler Technology Innovation Act of 1980* (Stevenson-Wydler), and the *Federal Technology Transfer Act of 1986* (FTTA of 1986) are the cornerstones of the legal foundations for federal agencies’ interpretation and implementation of their R&D management practices and technology transfer activities in pursuit of their respective missions [32]. Bayh-Dole allows non-profits and small businesses to keep title to inventions made using federal government funding. Enacted in December of 1980, Bayh-Dole—which made changes to U.S. Code (USC) title 35, i.e., the “Patents” chapter—also explicitly authorized federal agencies to “grant exclusive or partially-exclusive licenses to patents, patent applications, or other forms of protection obtained” (Codified as amended at 35 USC 200 et seq.). The importance of Bayh-Dole (PL 96–517) on innovation policy is well-documented in the existing literature and is prominently featured in numerous studies of federal technology transfer, academic entrepreneurship, and the commercialization of university-owned patents [33–36].

Prior to Bayh-Dole, the granting of an exclusive license was a lengthy and cumbersome endeavor, with specific requirements differing by the agency. In practice, exclusive licenses were almost never granted [37]. The elaborate process for the U.S. Navy, considered among the more forward-thinking agencies at the time [38], involved advertising the patent in three different publications (the U.S. Patent Office, the Federal Register, and at least one other publication of choice) for a period of at least six months, followed by another 60 day public notice of a prospective exclusive license [39]. Given the lengthy processes and bureaucratic hurdles, agencies would typically grant only *non-exclusive* licenses, which made a prospective licensee’s business decision of investing in federally-owned patented technologies far riskier. As a result, the government appeared to be hoarding around 30,000 unlicensed patents, and potentially useful and valuable new technologies were not being commercialized. Hence, prior to the enactment of Bayh-Dole, if one looked at patent licensing activity, federal technology transfer appeared to be at a complete standstill. For example, in 1976 alone, only about 150 patents were licensed by all federal agencies from over 2,000 issued patents [39]. With such a small fraction of patents actually being licensed for commercial use, proposed reforms recommended offering a degree of exclusivity in licensing through a unified and more streamlined process to accelerate the commercialization of unlicensed inventions [40, 41]. By explicitly authorizing federal agencies to exclusively license inventions, Bayh-Dole aimed to address this issue.

Although it is less well-known than Bayh-Dole, an equally important piece of legislation is Stevenson-Wydler (PL 96–480), which was enacted fifty-two days earlier and signed into law, also by President Carter. This law made changes to title 15, the “Commerce and Trade” chapter, and was focused on using the federal labs’ R&D capabilities and resulting technologies to more directly benefit citizens of the U.S. by moving those technologies to the private sector (Codified as amended at 15 USC 3701 et seq.). Of the two laws, Stevenson-Wydler is the only one to explicitly mention “technology transfer” and make technology transfer a codified mission of the federal labs by requiring each agency “strive where appropriate to transfer federally owned or originated technology to State and local governments and to the private sector [42].”

Agencies were legally bound to establish an Office of Research and Technology Applications (ORTA) at each lab, staff the office with at least one full-time professional, and devote not less than 0.5% of the agency’s R&D budget to support the technology transfer function. A waiver for the 0.5% budgetary requirement was built into the law, and essentially all of the agencies requested waivers; the requirement was dropped when the law was amended in 1986 [43–45]. From the beginning, the implementation of technology transfer legislation at federal agencies and national labs had its supporters [46, 47] and skeptics [48]. Part of the ongoing debate about the effectiveness of these policies and laws arose from the lack of a uniform, standardized set of metrics for consistently monitoring and measuring technology transfer performance across agencies, labs, and teams:

*“There is no single*, *obvious way to measure the success of tech transfer that everyone has somehow been missing*. *Metrics themselves should be seen as experimental*, *and their impact needs to be monitored*. *At the same time*, *metrics should not be altered lightly because stability is needed to make comparisons over time*.*”*

     RFI Response, Massachusetts Institute of Technology [49]

The enactment of Stevenson-Wydler required federal agencies to incorporate technology transfer activities directly into their respective missions and to allocate dedicated resources but did not adequately define appropriate metrics for measuring these activities across agencies. In the 1980s, several academic studies indicating that the U.S. faced a risky competitive decline in innovation, along with the growing recognition that many national labs had specialized facilities and equipment that could be leveraged to support innovation more broadly, prompted renewed attention on federal technology transfer [50–52]. In response, Congress passed the FTTA of 1986. Signed into law by President Reagan, the legislation amended Stevenson-Wydler in a number of noteworthy ways, including moving licensing from being handled centrally by agency managers to being handled by the inventing lab, establishing a Federal Laboratory Consortium, and most importantly, authorizing the use of *Cooperative Research and Development Agreements* (CRADAs) by the labs.

A CRADA is a contractual agreement between one or more federal laboratories and one or more non-federal entities “under which the Government, through its laboratories, provides personnel, services, facilities, equipment, intellectual property, or other resources with or without reimbursement (but not funds to non-Federal parties) and the non-Federal parties provide funds, personnel, services, facilities, equipment, intellectual property, or other resources toward the conduct of specified research or development efforts which are consistent with the missions of the laboratory” (15 USC 3710a). Shortly after the law came into effect, CRADAs experienced explosive growth and rapidly came to dominate the formal channels of federal technology transfer [22, 53–55].

In sum, the enactment of Bayh-Dole, Stevenson-Wydler, and the FTTA of 1986 established some of the guiding principles that remain influential today in shaping how federal agencies’ employees manage R&D activities. All three pieces of legislation address key issues regarding the ownership, transfer, and sharing of knowledge generated by federal labs. Bayh-Dole emphasized the importance of licensing out government-owned inventions. Stevenson-Wydler established the requirement that federal agencies explicitly incorporate technology transfer into their missions. The FTTA of 1986 broadly authorized resource-leveraging agreements in the form of CRADAs to facilitate collaboration with private sector partners. These laws collectively provide broad authorization and guidance to agencies conducting R&D on how to execute their mission with a focus on technology transfer.

### Conceptual framework

Prior research argues that an organization’s performance in managing R&D is influenced by: (1) the *types of problems* that the organization seeks to solve; and (2) the *forms of governance* that the organization intends to implement [56]. From the perspective of managers and policymakers, improving organizations’ technology transfer performance requires matching the governance form with the problem type [56–58]. Previous findings indicate that misaligned governance forms and problem types may be costly to organizations in terms of lost time and wasted resources and may be harmful in ways that are economically significant [59, 60]. Thus, the use of insufficient or unsuitable governance forms for problem-solving may inhibit or impede the inventive output and commercial activities that organizations ultimately seek to generate through producing new knowledge and engaging in R&D collaborations [61].

In the context of U.S. federal government technology transfer, we contend that evaluating the possible performance consequences of choosing governance forms that are congruent with problem types is especially important for producing useful knowledge [62–64]. The federal government and its individual executive branch agencies play a vital role in the production of scientific knowledge as a public good [65]. The core issue of allocation associated with knowledge [66, 67] as a public good is exacerbated by the fact that elected leaders, agency officials, taxpaying citizens, and private sector firms are all interested stakeholders in public sector R&D programs administered by a representative bureaucracy [68]. These divergent stakeholder groups may fail to reach a policy consensus because they are often driven by competing or conflicting political, social, and economic motivations for either supporting or opposing government R&D investment in creating national capacity for scientific problem-solving [69–71]. We argue that the frequent failure to achieve a broad-based policy consensus is due in part to a lack of standardized and consistent government-wide performance metrics for quantifying the value of technology transfer outcomes at the agency-level [29]. Furthermore, key constituencies such as taxpaying citizens, industry associations, advocacy groups, and media watchdogs may express valid concerns about only being able to observe the amount of financial resources allocated to and invested in public sector R&D activities without being able to observe any eventual commercial outcomes or actual economic impacts [72]. In other words, in the absence of observable outputs with quantifiable benefits, these constituencies tend to focus on the known costs of observable inputs. Hence, we contend that because the value of producing scientific knowledge and sharing it as a public good is usually difficult to ascertain [73], this makes actually introducing, reforming, replacing, or eliminating innovation programs difficult for public sector organizations such as federal agencies to initially justify and eventually operationalize [74].

To specifically address the challenge of formulating and executing evidence-based policies for managing government-funded innovation, our study attempts to develop managerially sound and empirically observable metrics that capture the implementation of certain salient governance forms within the distinct problem types encountered by federal agencies. We believe that these tools may help scholars, managers, and policymakers who are seeking further transparency and greater accountability when examining federal agencies’ management of public resources for R&D undertaken in the national interest.

It is generally easier to assess the potential commercial value of knowledge that is more applied and practical versus knowledge that is more basic and theoretical. For example, an optical sensor technology that can accurately detect faint objects from a great distance might enhance existing commercial products such as satellites. However, a new underlying theory that predicts the quantum effects of gravity on bending light from distant stars may be considerably more difficult to immediately use in commercial applications including satellites. In addition, knowledge that spans multiple scientific domains is more interdependent because it requires synthesizing discoveries across distinct disciplines. Greater knowledge interdependence is often associated with fundamental breakthroughs as opposed to incremental innovations. For example, nuclear physicists, computer scientists, and medical researchers from a range of national labs collaborated to originally prototype and develop medical imaging technologies that use radioisotopes to detect and treat cancerous tumors. Both dimensions–applied knowledge and interdependent knowledge–affect researchers’ abilities to solve scientific problems. Consistent with the prior literature, we posit that the scientific problems that organizations face may be characterized along two critical dimensions: the use of *applied knowledge* [75, 76] and the *interdependence of the knowledge* [77] required to solve the problem.

Hence, effectively solving a problem is dependent on knowing *how to navigate the interdependence* and knowing *where to search for the relevant knowledge* [78]. Compared to major R&D-intensive multinational corporations in the private sector, major R&D-intensive federal agencies are typically tasked by their leaders to engage in larger-scale and longer-term scientific endeavors that may have no immediate commercial value but that are nonetheless expected to be strategically important for the future of the country as a whole. FFRDCs and external (non-federal) R&D partners may possess unique resources and capabilities that are essential for commercializing scientific knowledge. This implies that utilizing FFRDCs and engaging in formal and informal R&D partnerships [79, 80] are potentially useful means for federal agencies to transfer technologies in fulfillment of their missions [81–83].

Leading R&D-intensive federal agencies–including the U.S. Department of Energy, the Department of Defense (DOD), the Department of Health and Human Services (HHS), the National Aeronautics and Space Administration (NASA), and the U.S. Department of Agriculture (USDA)–independently pursue their own portfolios of scientific research characterized by varying levels of applied versus basic knowledge and varying levels of knowledge interdependence across domains [84]. Analyzing similarities and differences across these agencies provides insight into how to navigate this interdependence and precisely where to search for the relevant knowledge. We argue that some of these similarities and differences are readily apparent in each agency’s decisions regarding the appropriate set of governance forms to apply in solving the types of scientific problems that are central to fulfilling their respective missions. These governance forms typically include a broad array of *knowledge sharing processes and structures*–from standardizing contracts, to forming alliances, to offering grants and prizes, to leveraging crowdsourced platforms, and building global user communities [85–88].

Unlike prior research on government and academic technology transfer, which focuses primarily on *protecting* proprietary technologies through patenting activities, here we examine the importance of engaging in external R&D *collaborations* (such as FFRDC utilization, traditional CRADAs, and other R&D collaborations) and *sharing* proprietary technologies through non-patent IP policies such as copyrights and MTAs [89, 90], and evaluate these agency-specific policies on performance measures. Because solving difficult scientific problems frequently requires the development of technical artifacts such as software, datasets, and materials as complementary resources and tools, we examine this aspect of knowledge sharing. In addition, “increased linkages to and knowledge flows from various external partners, particularly in uncertain environments, lead to improved innovation outcomes” [91].

“Although copyright can protect computer software, under the Government Works exception, the U.S. Government is prohibited from claiming copyright in the United States in any works prepared by officers or employees of the Federal Government in the course of their official duties” [49]. “MTAs may apply to anything from materials that are simply under the control of the originator but have no formal intellectual property rights attached to them to proprietary materials protected by patents and trade secrets” [92]. Defining policies for the use of copyrights and MTAs presents an interesting dilemma for managers of federal agencies. On the one hand, potential external R&D partners and prospective licensees often need access to accompanying software, datasets, materials, and associated know-how to be able to further develop and possibly commercialize technologies transferred from federal labs. On the other hand, if the copyright status or property transfer rights of the software, datasets, materials, or know-how is unclear or infeasible due to the agency’s interpretation and implementation of federal statutes, this may hinder the subsequent use of these essential technical artifacts in the development of commercial applications in the private sector.

In sharp contrast to the emerging set of studies on federal technology transfer that utilize only supply-side performance metrics [28], we propose the use of demand-side metrics as well [93, 94]. For example, previous research recommends measuring the filing ratio, transfer rate, and licensing success rate to evaluate the effectiveness of federal technology transfer efforts [29, 30]. A key limitation of all of these metrics is that they represent the potential market readiness of the technologies solely from the *selling producer’s perspective*. We attempt to overcome this limitation by extending the relevant metrics to include the licensing income generated and the degree of portfolio exclusivity associated with income bearing licenses. We contend that licensing income and portfolio exclusivity represent actual market acceptance of the technologies from the *paying customer’s perspective*. In other words, filing ratio, transfer rate, and licensing success rate reflect an agency’s ability to prudently manage and convert its technologies into accessible artifacts, while licensing income and portfolio exclusivity reflect actual customers’ market adoption of the technical artifacts generated by the agency. In keeping with the emphasis of recent administrations on Lab to Market (Lab2M) initiatives across the entire U.S. federal government, we believe a balanced usage of both supply-side and demand-side performance metrics is warranted. A holistic appraisal of supply- and demand-side metrics may help stakeholders better understand technology transfer performance and drive a policy consensus.

### Managing federal technology transfer performance

The historical context and conceptual framework we proposed above suggest that there is a set of practical and actionable ideas that should be explored empirically to provide federal technology managers with an initial sense of the potential direction and magnitude of the possible effects of key innovation management decisions under their control. For example, when allocating their annual R&D budgets and aligning these budgets with their missions, federal technology managers are empowered to determine their respective agencies’ utilization of FFRDCs. Instead of using classical contracts or CRADAs, greater reliance on FFRDCs for conducting R&D might plausibly reduce supply-side technology performance while boosting demand-side performance. This is because the generation of key knowledge artifacts becomes more decentralized and more distributed, though still without a commercial sector nexus, when it is shifted from agency-operated sites to contractor-operated FFRDCs. However, this same shift may advance the technologies closer to commercialization by mitigating scientific or technical risk, which may enhance demand-side performance.

In addition, greater use of formal R&D agreements such as portfolios of CRADAs [95] as well as informal R&D partnerships, which feature public sector agency and private sector firm (government-industry) relationships and interactions, may have different effects on supply-side performance. Partnership agreements such as CRADAs may facilitate improved filing ratios, transfer rates, and licensing success rates by clearly specifying partnership terms and conditions and IP rights in a structured and consistent manner that is straightforward for external R&D partners to grasp and implement. In contrast, non-standardized forms of external R&D partnerships such as one-off custom collaborative agreements may reduce supply-side performance by constraining technology managers’ options for IP protection [96] and increasing the complexity of licensing arrangements.

The implementation of non-patent IP policies varies considerably across federal agencies. If an agency’s innovation activities generate substantial amounts of software, data, and expertise that is useful and valuable to the private sector, then non-patent IP policies such as copyright agreements are directly relevant to the agency’s technology transfer performance. Or, if an agency’s innovation activities involve unique or difficult to obtain proprietary materials such as biological samples or chemical compounds that are of commercial importance, then MTAs are also directly relevant to the agency’s performance. Differences in the adoption of copyright agreements and MTAs may account for observed differences between supply-side and demand-side metrics.

## Methodology and data

### Data sources and collection procedures

Our sample timeframe is from 1999–2016 and our unit of analysis is at the federal agency level. Under the Technology Transfer Commercialization Act of 2000 (TTCA of 2000, PL 106–404), U.S. federal agencies are required to report certain measurements annually. Under this statute (15 USC 3710(f)(2)(B)), the specific parameters are: (1) the number of patent applications filed; (2) the number of patents received; (3) the number of fully-executed licenses that received royalty income in the preceding fiscal year, categorized by whether they are exclusive, partially-exclusive, or non-exclusive; (4) the time elapsed from the date the license was requested by the licensee in writing to the date the license was executed; (5) the total earned royalty income; (6) what disposition was made of the income described in the clause; (7) the number of licenses terminated for cause; and (8) any other parameters or discussion that the agency deems relevant or unique to its practice of technology transfer (in practice, (4) and (6) are not actually reported by U.S. federal agencies). In addition, agencies also report the number of invention disclosures, as well as the number of CRADAs.

We use variables collected from three online databases managed by the U.S. federal government. The National Science Foundation (NSF) maintains two of these databases: (1) the National Center for Science and Engineering Statistics (NCES) database; and (2) the FFRDC R&D Survey. The remaining database is from the National Institute of Standards and Technology (NIST) Annual Technology Transfer Reports managed by the U.S. Department of Commerce (DOC).

NIST Annual Technology Transfer Reports contain information about CRADAs, invention disclosures and patenting, portfolio profiles of active licenses, and income from licensing. We downloaded and gathered our data from the FY 2003–2019 annual reports, which covers technology transfer activities over the eighteen-year period of 1999–2016, inclusive.

The FFRDC R&D Survey is the main source of information on R&D expenditures at FFRDCs in the U.S. These annual surveys categorize R&D spending by: (1) source of funds (federal, state and local, business, nonprofit, or other); (2) federal agency source; (3) type of R&D (basic research, applied research, or development); (4) type of costs (salaries, software, equipment, subcontracts, and other direct or indirect costs); and (5) total operating budget.

The NCES database provided statistical data about the breakdown of R&D expenditures at FFRDCs (university-administered, nonprofit-administered, and industry-administered) by the source of funds. Specifically, NCES indicates the R&D funds allocated by the federal government to state and local government, businesses, nonprofit organizations, or other federal and non-federal entities.

### Variables

We use the NIST, NSF-NCES, and NSF-FFRDC R&D databases to construct and compute the following dependent, explanatory, and control variables, as described below.

#### Dependent variables

Our analysis spans five primary dependent variables that represent technology transfer activities managed by federal agencies [97]. The first set of dependent variables serves as supply-side metrics for evaluating technology transfer outcomes. The *Filing Ratio* is the ratio of the number of patent applications divided by the total number of new invention disclosures in a given fiscal year [29]. *Transfer Rate* is the ratio of the number of newly granted patent licenses over the total number of filed patent applications in a given fiscal year [29]. *Licensing Success Rate* is the number of licenses granted out of the total number of invention disclosures received by an agency’s Technology Transfer Office (TTO) in a given fiscal year [30].

The second set of dependent variables captures demand-side metrics for evaluating the technology transfer performance of federal agencies. *License Income (log)* is the base 10 logarithm of the annual dollar amount of licensing income compiled and communicated by NIST in a given fiscal year [97, 98]. *Portfolio Exclusivity* is the total annual amount of active exclusive licenses divided by total active income bearing licenses communicated by NIST in a given fiscal year [97, 99].

#### Explanatory variables

Our two sets of explanatory variables represent federal agencies’ R&D collaborations and non-patent IP policies. The first set of explanatory variables measure the extent of federal agencies’ external R&D collaborations. *FFRDC Utilization (%)* is the percentage of FFRDCs utilized by a given agency, in a given year. It is important to note that some agencies fund R&D projects conducted by FFRDCs that are primarily aligned with other agencies. For example, the DOD funds research at multiple FFRDCs associated with the DOE. There are many types of CRADAs, and here we focus on those that formalize a substantive collaboration, the traditional CRADAs. *Traditional CRADAs (log)* is the base 10 logarithm of an agency’s number of traditional CRADAs. *Other R&D Collaborations (log)* is the base 10 logarithm of the number of other collaborative R&D relationships that are not part of traditional CRADAs. These measures capture different dimensions of the size and scale of an agency’s R&D partner portfolio.

Our second set of explanatory variables defines federal agencies’ adoption of policies for non-patented IP. *Copyright Policy* is a binary variable, which is 1 if the federal agency adopted copyright IP policy as a mechanism for technology transfer, as disclosed by NIST and equals 0 otherwise. *Materials Transfer Policy* is s a binary variable, which is 1 if the federal agency adopted MTAs as a mechanism for technology transfer, as disclosed by NIST, and equals 0 otherwise.

#### Control variables

To account for alternative explanations, we include a set of control variables at the U.S. federal agency level that may plausibly influence technology transfer activities. *R&D Asset Intensity (%)* is the percentage of total annual R&D obligations allocated to R&D plants in the form of fixed assets such as specialized facilities. *R&D Applied Knowledge (%)* is the dollar-weighted percentage index of R&D expenditure in Applied R&D activities as a percentage of the total R&D budget disclosed to NSF-NCSES in a given fiscal year. The following control variables account for agencies’ allocation of R&D to other entities. *R&D to Universities (%)* is the percentage of total annual R&D obligations performed by academic institutions (universities and colleges, excluding FFRDCs). *R&D to State and Local Govt*. *(%)* is the percentage of total annual R&D obligations performed by state and local governments.

### Model specification

Because of the nature of our data (repeated observations across agencies over multiple years) we follow a panel data methodology. To deal with additional unobserved heterogeneity, we use random effects panel regressions using the *xtreg* command in Stata 15 statistical software [100]. The large chi-square value from Breusch-Pagan test reveals heteroscedasticity; thus we run our models with the ‘vce (robust)’ option, which computes robust standard errors and accounts for clustered observations by agency [101].

## Results

### Descriptive statistics and correlations

Table 1 below lists the means, standard deviations, minimum, maximum, and correlations among the variables in our models for the full sample (FY 1999–2016). The average *Filing Ratio* of federal agencies in our sample is 61%, which is consistent with the university industry-wide benchmark [102]. The mean value for *Transfer Rate*, a measure of effectiveness, over the 18-year period is about 75%. This is significantly higher than the 42% industry-wide norm [29] but is driven by an aging success from the Department of Commerce (see DOC Transfer Rates 18yrs and 5yrs in Table 3). The average *Licensing Success Rate*, a measure of efficiency, is 34%, which is higher than the 25% industry-wide benchmark, and reflects the efficiency of a technology transfer at federal agencies [30]. We note that our sample is a longitudinal panel spanning fiscal years 1999–2016; hence our benchmarks may vary from the results reported in the previous cross-sectional studies cited in the literature. Here too, the number is driven by historical success, also out of the DOC. The mean values for *FFRDC Utilization (%)*, *Traditional CRADAs (log)*, and *Other R&D Collaborations (log)* are 19.34%, 1.96, and 1.25, respectively, which indicate the average strength of R&D collaborations at federal agencies between 1999 and 2016. In terms of non-patent IP policies, about 29% of agencies adopted a non-patent invention policy (*Copyright Policy*) and about 48% of them adopted MTAs (*Materials Transfer Policy*).

**Table 1:** Descriptive statistics and correlations (full sample 1999–2016).

	Variable	Mean	S.D.	Min	Max	1	2	3	4	5	6	7	8	9	10	11	12	13	14
	**Dependent Variables**																		
1	Filing Ratio	0.61	0.46	0	3.67	1.00													
2	Transfer Rate	0.75	2.48	0	23.38	-0.12	1.00												
3	Licensing Success Rate	0.34	0.81	0	5.93	0.05	**0.92**	1.00											
4	License Income (log)	5.65	2.10	0	8.21	-0.03	-0.01	-0.01	1.00										
5	Portfolio Exclusivity	0.29	0.27	0	1.00	-0.10	0.11	0.09	0.05	1.00									
	**Explanatory Variables**																		
	***R&D Collaborations***																		
6	FFRDC Utilization (%)	19.34	22.99	0	85.71	-0.01	-0.08	-0.11	**0.31**	**-0.24**	1.00								
7	Traditional CRADAs (log)	1.96	0.93	0	3.44	**0.15**	0.01	0.02	**0.36**	0.01	**0.46**	1.00							
8	Other R&D Collaborations (log)	1.25	1.56	0	4.21	**-0.15**	**0.17**	0.14	0.10	**0.42**	-0.13	**-0.22**	1.00						
	***Non-Patent IP Policies***																		
9	Copyright Policy	0.29	0.45	0	1.00	**-0.30**	**0.21**	**0.16**	**0.26**	0.01	0.08	**-0.15**	**0.34**	1.00					
10	Materials Transfer Policy	0.48	0.50	0	1.00	-0.06	**0.15**	**0.16**	**0.29**	**0.26**	0.12	**0.49**	**0.20**	**-0.18**	1.00				
	**Control Variables**																		
11	R&D Asset Intensity (%)	5.77	9.44	0	54.88	**-0.19**	0.00	-0.04	**-0.31**	-0.13	0.08	-0.10	**0.18**	**0.31**	**-0.23**	1.00			
12	R&D Applied Knowledge (%)	53.49	23.43	18.53	97.02	**0.25**	-0.01	-0.01	-0.14	-0.01	**0.24**	-0.02	-0.10	**-0.23**	**-0.19**	-0.07	1.00		
13	R&D to Universities (%)	15.47	15.42	0	57.81	0.12	0.09	**0.18**	**0.38**	-0.07	-0.08	0.13	0.13	-0.14	**0.36**	-0.11	**-0.43**	1.00	
14	R&D to State and Local Govt. (%)	1.93	5.02	0	30.93	0.09	-0.06	-0.05	**-0.40**	0.05	-0.12	**-0.30**	-0.08	**-0.18**	**-0.30**	-0.11	-0.02	-0.13	1.00

N = 127; p<0.05 in bold

As shown in Table 1, the largest significant correlation appears to be 0.92, which is between *Licensing Success Rate* and *Transfer Rate*. This is expected since both contain the number of licenses in the numerator, with the difference in the denominator being either disclosures (LSR) or filed patent applications (TR). The high correlation shows the historical patent-centric model of licensing and is not an issue, since both dependent variables are not used in the same regression models. We check the variance inflation factor (VIF) for all variables in our models. We find that average VIF for our variables is 1.63 and no variable exceeds 2.08, indicating that multicollinearity is not an issue in any of our models. Table 2 below shows the descriptive statistics for the last 5 years of our sample (FY 2012–2016, inclusive).

**Table 2:** Descriptive statistics (5 Years 2012–2016).

	Variable	Mean	S.D.	Min	Max
	**Dependent Variables**				
1	Filing Ratio	0.55	0.36	0	1.80
2	Transfer Rate	0.40	1.09	0	8.00
3	Licensing Success Rate	0.18	0.28	0	1.33
4	License Income (log)	5.78	1.71	0	8.21
5	Portfolio Exclusivity	0.31	0.32	0	1.00
	**Explanatory Variables**				
	***R&D Collaborations***				
6	FFRDC Utilization (%)	19.91	23.97	0	83.33
7	Traditional CRADAs (log)	2.11	0.92	0	3.43
8	Other R&D Collaborations (log)	1.85	1.48	0	4.21
	***Non-Patent IP Policies***				
9	Copyright Policy	0.27	0.45	0	1.00
10	Materials Transfer Policy	0.46	0.50	0	1.00
	**Control Variables**				
11	R&D Asset Intensity (%)	5.91	11.43	0	54.88
12	R&D Applied Knowledge (%)	52.29	23.44	23.86	97.02
13	R&D to Universities (%)	14.69	15.22	0	56.14
14	R&D to State and Local Govt. (%)	1.96	5.17	0	21.53

Table 3 presents the average summary of our variables broken down by 10 agencies in the fiscal years 1999–2016 and 2012–2016. We elaborate on the interpretation of the agency-level summary table (Table 3) in the discussion section of the paper.

**Table 3:** Data summary of measures.

**Agency**	**DOC**		**DOD**		**DOE**		**DOI**		**DOT**	
	**18 Yrs**	**5 Yrs**	**18 Yrs**	**5 Yrs**	**18 Yrs**	**5 Yrs**	**18 Yrs**	**5 Yrs**	**18 Yrs**	**5 Yrs**
Filing Ratio	0.516	0.506	0.775	1.002	0.531	0.572	0.965	0.700	0.831	0.295
Transfer Rate	4.080	0.349	0.080	0.050	0.214	0.173	0.371	0.217	0.190	0.000
Licensing Success Rate	1.451	0.167	0.058	0.052	0.111	0.097	0.310	0.172	0.236	0.000
License Income (log)	5.414	5.290	7.041	7.017	7.577	7.589	4.828	4.943	3.101	4.112
Portfolio Exclusivity	0.462	0.549	0.287	0.267	0.083	0.054	0.291	0.398	0.325	0.200
FFRDC Utilization (%)	7.804	5.238	75.529	81.429	39.418	38.571	0.661	0.476	13.624	6.190
Traditional CRADAs (log)	2.180	2.365	3.282	3.295	2.839	2.870	1.450	1.506	1.194	1.119
Other R&D Collaborations (log)	3.026	3.483	0.984	2.269	0.000	0.000	0.820	2.488	0.607	1.568
R&D Asset Intensity (%)	11.732	14.392	0.293	0.256	9.707	6.962	0.846	0.774	2.833	2.912
R&D Applied Knowledge (%)	50.592	46.237	93.344	93.025	28.341	25.011	60.384	59.197	50.716	51.723
R&D to Universities (%)	16.321	17.104	4.223	3.948	9.703	9.201	9.292	7.905	7.557	8.228
R&D to State and Local Govt. (%)	0.681	0.415	0.094	0.047	0.084	0.122	1.680	2.012	15.170	17.899
Total R&D (Million)	1283.000	1449.000	59800.000	61870.000	9607.000	11600.000	680.200	778.200	785.100	901.100
	**Non-Patent IP Policies (+/-)**									
Copyright Policy	Yes		No		Yes		No		No	
Materials Transfer Policy	Yes		Yes		No		No		No	
**Agency**	**EPA**		**HHS**		**NASA**		**USDA**		**VA**	
	**18 Yrs**	**5 Yrs**	**18 Yrs**	**5 Yrs**	**18 Yrs**	**5 Yrs**	**18 Yrs**	**5 Yrs**	**18 Yrs**	**5 Yrs**
Filing Ratio	0.980	0.794	0.639	0.706	0.128	0.084	0.726	0.722	0.227	0.171
Transfer Rate	0.835	1.830	0.886	0.779	0.357	0.406	0.266	0.199	0.388	0.161
Licensing Success Rate	0.442	0.579	0.536	0.553	0.045	0.034	0.186	0.143	0.097	0.017
License Income (log)	5.159	5.593	8.004	8.139	6.540	6.472	6.257	6.689	4.728	5.499
Portfolio Exclusivity	0.198	0.242	0.078	0.079	0.336	0.061	0.590	0.716	0.406	0.787
FFRDC Utilization (%)	2.381	8.571	28.042	30.952	19.709	17.143	0.132	0.000	0.000	0.000
Traditional CRADAs (log)	1.777	1.747	2.373	2.473	0.212	0.223	2.316	2.285	1.986	3.211
Other R&D Collaborations (log)	0.000	0.000	0.478	1.720	3.258	3.307	3.404	4.164	0.000	0.000
R&D Asset Intensity (%)	1.794	1.095	1.177	0.465	9.877	0.841	4.098	0.646	0.053	0.000
R&D Applied Knowledge (%)	94.194	96.147	23.988	25.411	56.584	42.122	35.637	34.311	40.954	38.531
R&D to Universities (%)	15.104	13.694	56.009	55.707	10.417	8.581	30.172	30.494	0.000	0.000
R&D to State and Local Govt. (%)	0.730	0.000	0.653	0.588	0.775	0.033	0.318	0.388	0.000	0.000
Total R&D (Million)	559.400	536.600	28490.000	30890.000	9112.000	11200.000	2225.000	2244.000	486.300	639.900
	**Non-Patent IP Policies (+/-)**									
Copyright Policy	No		No		Yes		No		No	
Materials Transfer Policy	No		Yes		No		Yes		Yes	

### Summary of results

We present the results of our study and models for our analyses of federal agency technology transfer performance in Table 4. As shown in Table 4, a 1% unit increase in the *FFRDC Utilization (%)* is associated with a 4.76% decrease (Model 2: β = -0.0476, p<0.01) in *Transfer Rate*, 1.89% decrease (Model 3: β = -0.0189, p<0.01) in *Licensing Success Rate*, 3.15% increase (Model 4: β = 0.0315, p<0.01) in *License Income (log)*, and 0.44% decrease (Model 5: β = -0.0044, p<0.01) in *Portfolio Exclusivity*. In Table 4 (Model 1), we see that the coefficient for the *FFRDC Utilization (%)* is negative (β = -0.0012), but not significant for the *Filing Ratio*.

**Table 4:** The effects of R&D collaborations and non-patent IP policies on federal agency technology transfer performance metrics.

Dependent Variable	Model 1	Model 2	Model 3	Model 4	Model 5
	Supply-Side	Supply-Side	Supply-Side	Demand-Side	Demand-Side
	Coefficients	Coefficients	Coefficients	Coefficients	Coefficients
	5-Yr Lag	5-Yr Lag	5-Yr Lag	5-Yr Lag	5-Yr Lag
	Filing Ratio	Transfer Rate	Licensing Success Rate	License Income (log)	Portfolio Exclusivity
**Explanatory Variables**					
***R&D Collaborations***					
FFRDC Utilization (%)	-0.0012	-0.0476***	-0.0189***	0.0315***	-0.0044***
	(0.0023)	(0.0132)	(0.0062)	(0.0057)	(0.0008)
Traditional CRADAs (log)	0.2353***	0.3940**	0.2046***	-0.0945	0.0814**
	(0.0480)	(0.1795)	(0.0716)	(0.1076)	(0.0390)
Other R&D Collaborations (log)	0.0507***	-0.2965*	-0.1645	-0.1214**	0.0639***
	(0.0196)	(0.1591)	(0.1077)	(0.0612)	(0.0135)
***Non-Patent IP Policies***					
Copyright Policy	-0.3651**	4.6061***	1.6179**	0.3971	-0.2100***
	(0.1619)	(1.6863)	(0.6650)	(0.4739)	(0.0401)
Materials Transfer Policy	-0.3312***	2.0366***	0.7156**	-0.1190	0.2036***
	(0.1216)	(0.6391)	(0.3522)	(0.4227)	(0.0516)
**Control Variables**					
R&D Asset Intensity (%)	0.0016	-0.1370	-0.0400	0.0082	0.0094***
	(0.0019)	(0.1128)	(0.0294)	(0.0060)	(0.0025)
R&D Applied Knowledge (%)	0.0021	0.0378***	0.0144**	-0.0050	-0.0009
	(0.0020)	(0.0110)	(0.0058)	(0.0069)	(0.0008)
R&D to Universities (%)	0.0042	0.0229	0.0124*	0.0388***	-0.0080***
	(0.0028)	(0.0145)	(0.0067)	(0.0070)	(0.0009)
R&D to State and Local Govt. (%)	0.0034	0.1043**	0.0462**	-0.1112***	0.0158***
	(0.0064)	(0.0475)	(0.0214)	(0.0194)	(0.0029)
Constant	0.1945	-3.0940***	-1.2226**	5.5924***	0.2793***
	(0.2124)	(1.0731)	(0.5330)	(0.7052)	(0.1034)
Observations	127	128	127	130	130
Gaussian Wald Chi Square	6893	1347	3277	40907	61799
R Square (within)	0.0339	0.0713	0.0896	0.0313	0.133
R Square (between)	0.832	0.904	0.816	0.925	0.950
R Square (overall)	0.411	0.256	0.289	0.664	0.551

Note: *** p<0.01,

** p<0.05,

* p<0.1; Standardized coefficients are reported; Robust standard errors in parentheses

As shown in Table 4, we find that one order of magnitude increase in *Traditional CRADAs (log)* in federal agencies is associated with a 23.52% increase (Model 1: β = 0.2353, p<0.01) in *Filing Ratio* (indicating less active management), a 39.40% increase (Model 2: β = 0.3940, p<0.05) in *Transfer Rate*, a 20.46% increase (Model 3: β = 0.2046, p<0.01) in *Licensing Success Rate*, and an 8.14% increase (Model 5: β = 0.0814, p<0.05) in *Portfolio Exclusivity*. In Table 4 (Model 4), we see that the coefficient for the *Traditional CRADAs (log)* is negative (β = -0.0945), but is not significant for *License Income (log)*.

A one order of magnitude increase in *Other R&D Collaborations (log)* is associated with a 5.07% increase (Model 1: β = 0.0507, p<0.01) in *Filing Ratio*, a 29.65% decrease (Model 2: β = -0.2965, p<0.1) in *Transfer Rate*, a 12.14% decrease (Model 4: β = -0.1214, p<0.05) in *License Income (log)*, and a 6.39% increase (Model 5: β = 0.0639, p<0.01) in *Portfolio Exclusivity*. In Table 4 (Model 3), we see that the coefficient for the *Other R&D Collaborations (log)* is negative (β = -0.1645), but is not significant for *Licensing Success Rate*.

In terms of non-patent IP policies, establishing a non-patent invention policy (*Copyright Policy*) is broadly associated with improved metrics, such as a 36.51% decrease (Model 1: β = -0.3651, p<0.05) in *Filing Ratio* (reflecting more prudent management), a 460.61% increase (Model 2: β = 4.6061, p<0.01) in *Transfer Rate*, a 161.79% increase (Model 3: β = 1.6179, p<0.05) in *Licensing Success Rate*, and a 21% decrease (Model 5: β = -0.2100, p<0.01) in *Portfolio Exclusivity*. In Table 4 (Model 4), we see that the coefficient for the *Copyright Policy* is positive (β = 0.3971), but is not significant for *License Income (log)*.

As shown in Table 4, adopting MTAs (*Materials Transfer Policy*) is associated with a 33.12% decrease (Model 1: β = -0.3312, p<0.01) in *Filing Ratio*, a 203.66% increase (Model 2: β = 2.0366, p<0.01) in *Transfer Rate*, a 71.56% increase (Model 3: β = 0.7156, p<0.05) in *Licensing Success Rate*, and a 20% increase (Model 5: β = 0.2036, p<0.01) in *Portfolio Exclusivity*. In Table 4 (Model 4), we see that the coefficient for the *Materials Transfer Policy* is negative (β = -0.1190), but not significant for *License Income (log)*.

In Table 5, we report the results of the interaction effects of non-patent IP policies on federal agency technology transfer performance. As shown in Table 5, for the supply-side metrics, the interaction term between *Copyright Policy* and *Materials Transfer Policy* is positive and significant for *Filing Ratio* (Model 1: β = 0.6381, p<0.05), *Transfer Rate* (Model 2: β = 4.2867, p<0.01), and *Licensing Success Rate* (Model 3: β = 1.7917, p<0.01). For the demand-side metrics, the interaction term between *Copyright Policy* and *Materials Transfer Policy* is negative and significant for *License Income (log)* (Model 4: β = -3.4228, p<0.01) and positive (β = 0.1994), but is not significant for *Portfolio Exclusivity*.

**Table 5:** The interaction effects of non-patent IP policies on federal agency technology transfer performance metrics.

Dependent Variable	Model 1	Model 2	Model 3	Model 4	Model 5
	Supply-Side	Supply-Side	Supply-Side	Demand-Side	Demand-Side
	Coefficients	Coefficients	Coefficients	Coefficients	Coefficients
	5-Yr Lag	5-Yr Lag	5-Yr Lag	5-Yr Lag	5-Yr Lag
	Filing Ratio	Transfer Rate	Licensing Success Rate	License Income (log)	Portfolio Exclusivity
**Explanatory Variables**					
***Non-Patent IP Policies***					
Copyright Policy	-0.6898***	1.0857	0.1050	2.3564***	-0.2727**
	(0.1842)	(1.2638)	(0.3709)	(0.6888)	(0.1227)
Materials Transfer Policy	-0.4546**	-0.1773	-0.2169**	1.4175***	0.1992**
	(0.2201)	(0.3971)	(0.0936)	(0.3341)	(0.0918)
Copyright Policy X Materials Transfer Policy	0.6381**	4.2867***	1.7917***	-3.4228***	0.1994
	(0.2481)	(0.4811)	(0.1158)	(0.6899)	(0.1271)
**Control Variables**					
R&D Asset Intensity (%)	0.0012	-0.1231	-0.0376	-0.0018	0.0118***
	(0.0014)	(0.1162)	(0.0334)	(0.0073)	(0.0038)
R&D Applied Knowledge (%)	-0.0017	0.0041	0.0002	0.0136*	-0.0023*
	(0.0026)	(0.0062)	(0.0013)	(0.0071)	(0.0013)
R&D to Universities (%)	0.0049	0.0093	0.0062	0.0380***	-0.0073***
	(0.0032)	(0.0077)	(0.0038)	(0.0092)	(0.0028)
R&D to State and Local Govt. (%)	-0.0249***	0.0035	0.0005	-0.0361***	0.0123***
	(0.0021)	(0.0274)	(0.0093)	(0.0027)	(0.0031)
Constant	1.0239***	0.4014	0.3439***	3.7645***	0.4824***
	(0.2162)	(0.4596)	(0.1074)	(0.5747)	(0.1339)
Observations	127	128	127	130	130
Gaussian Wald Chi Square	470.4	3588	119250	790.0	268.4
R Square (within)	0.0807	0.0552	0.0355	0.0360	0.122
R Square (between)	0.444	0.961	0.982	0.847	0.617
R Square (overall)	0.260	0.258	0.296	0.646	0.378

Note: *** p<0.01,

** p<0.05,

* p<0.1; Standardized coefficients are reported; Robust standard errors in parentheses

## Discussion

With the caveat that we assumed consistent and accurate reporting across the agencies, our analysis underscores the value that a broad view of technology transfer brings to a federal agency. Specifically, our model indicates that agencies’ technology transfer success, as judged by the licensing-focused metrics of transfer rate and licensing success rate, is typically improved when those agencies have considered and implemented diverse approaches to disseminating non-patented proprietary artifacts, including data, software, and materials. In general, those agencies also have reduced filing ratios, indicating a higher level of acumen and rigor in making and supporting technology transfer decisions and activities. They also have a higher number of traditional CRADAs as normalized to their R&D budgets, indicating a more open approach to technology transfer.

### The importance of non-patent policies

Technology transfer has historically focused on inventions, and specifically patentable inventions, even as the word ‘invention’ remained undefined in Stevenson-Wydler. It was not until 1986 and PL 99–502 that Congress made it clear their view of ‘inventions’ referred to “any invention or discovery which is or ***may*** [emphasis added] be patentable or otherwise protected under title 35, United States Code, or any novel variety of plant which is or may be protectable under the Plant Variety Protection Act (7 U.S.C. 2321 et seq.).” Setting aside the circular nature of the definition, a lot of things *may* be patentable under U.S. patent law, at least back in 1986. However, the technology areas that lend themselves to patent protection are becoming fewer, and the protection itself available through patents is becoming narrower [103]. Thus, the trends in technology licensing have naturally evolved to include (if not increasingly rely on) other forms of IP, even as scholarly attention still concentrates on patents due to their ease of access [104]. Unfortunately, despite how easily quantifiable patents are, few sources document trade secrets and know-how licenses, and the extent to which federal laboratories participate in those types of transactions is not clear.

However, some agencies have promulgated non-patent IP policies related to technology transfer, such as copyright and MTA policies [49], indicating a broader view of technology transfer. There appears to be a strong association between improved agency performance and the implementation of these non-patent technology transfer mechanisms. Agency performance is ranked in Table 6, and each of these factors is associated with improved transactional count metrics, indicating more opportunities to leverage resources, accelerate R&D, and boost commercialization efforts. For example, the establishment of a copyright policy (at DOC, DOE, and NASA) is associated with exercising a more prudent use of patenting resources as measured by the filing ratio, even if copyrights have not been historically available to federal employees (See: 17 USC 105; copyright protection is generally not available to Government Owned Government Operated laboratories (GOGOs) but is available to Government Owned Contractor Operated laboratories (GOCOs)). There is also an association between employing a copyright policy and offering more non-exclusive licenses (see Table 4). Here, it is interesting to note that the three agencies mentioned above take different policy approaches to licensing software. DOC, which includes NIST as well as the National Oceanic and Atmospheric Administration (NOAA), does not appear to ‘license’ software and datasets, preferring to place it in the public domain, and so the non-exclusive license counts reported in the annual reports presumably do not include software [105]. This may be due to DOC labs being mainly government owned and government operated, making copyright protection generally unavailable. NASA applies a similar approach, while DOE licenses software from the contractor-managed national labs [106]. Enacting another non-patent policy—a material transfer policy—is similarly associated with improved performance metrics. Three of the top five performers for transfer rate have MTA policies. In fact, for all of the performance metrics shown in Table 6, one thing is common among the majority of top agencies: the official adoption of defined non-patent IP policies.

**Table 6:** Agency technology transfer performance.

Performance Rankings	SUPPLY-SIDE						DEMAND-SIDE		R&D COLLABORATION	
	Filing Ratio		Transfer Rate		Licensing Success Rate		R&D Budget/License Income		R&D Budget/CRADAs	
	18 Yrs	5 Yrs	18 Yrs	5 Yrs	18 Yrs	5 Yrs	18 Yrs	5 Yrs	18 Yrs	5 Yrs
1	NASA	NASA	DOC	EPA	DOC	EPA	DOE	HHS	VA	VA
2	VA	VA	HHS	HHS	HHS	HHS	HHS	DOE	DOC	DOC
3	DOC	DOT	EPA	NASA	EPA	DOI	USDA	USDA	EPA	EPA
4	DOE	DOC	VA	DOC	DOI	DOC	NASA	EPA	USDA*	USDA*
5	HHS	DOE	DOI	DOI	DOT	USDA	EPA	VA	DOE	DOE
6	USDA	DOI	NASA	USDA	USDA	DOE	DOC	NASA	DOI	DOI
7	DOD	HHS	USDA	DOE	DOE	DOD	DOD	DOD	DOD	DOD
8	DOT	USDA	DOE	VA	VA	NASA	VA	DOC	DOT	DOT
9	DOI	EPA	DOT	DOD	DOD	VA	DOI	DOI	HHS*	HHS*
10	EPA	DOD	DOD	DOT	NASA	DOT	DOT	DOT	NASA*	NASA*

* These agencies have additional statutory authority beyond CRADAs

Most agencies tend to use the CRADA authority to execute various agreement types, including MTAs, non-disclosure agreements, and traditional CRADAs [107]. However, HHS as well as USDA have long had distinct statutory authorities to transfer materials. For example, the original Public Health Service (PHS) Act of 1944 explicitly highlighted open and coordinated research (See PL 78–410). As amended, it broadly enables cooperative research across the full PHS (See: 42 USC 241), as well as explicitly authorizes the transfer of ‘substances’ from its largest intramural component (See: 42 USC 282(c))—the National Institutes of Health (NIH).

This World War II-era law seeded a culture of cooperative research, and in an attempt to streamline the sharing of biomaterials among universities and non-profits, NIH proposed to use an efficient Universal Biological Material Transfer Agreement (UBMTA) in a notice in the Federal Register (60 FR 12771–12775). However, they explicitly recognized not all agencies may have the same ability to execute a stand-alone UBMTA implementing document and that *other agencies* may need to alternatively execute a CRADA due to a perceived lack of a legal authority. *Therefore*, *either through explicit legal authorities or through permissive interpretation of existing authorities*, *agencies positioned for greater success are those able to expand their interpretations of both a) which assets are transferable and b) the statutory mechanisms available*.

### The importance of open innovation

CRADAs were authorized under the FTTA of 1986. The importance of CRADAs has endured and grown, and there continue to be far more CRADAs (or similar partnership agreements) than licenses executed by the individual laboratories and across all agencies [29]. However, even as their importance (by the numbers) far outweighs that of licenses, the emphasis on licensing lingers, and a recent report by the Government Accountability Office attempted to prescribe actions to increase licensing activity [108].

Several years prior to the enactment of the TTCA of 2000 and the required annual agency reports, a survey of industry leaders was conducted to ascertain what, in fact, industry wanted from the federal labs. The thinking was that rather than asking policymakers and academics for answers, it would make sense to seek real-world perspectives from the very people responsible for conducting the practical commercial application side of technology transfer: industry partners. Industry was found to view the very outputs Congress focuses on as actually providing minimal value, with more value stemming from contract and cooperative R&D activities, as well as idea transfer vs. technology transfer *per se* [31]. In this regard, industry leaders saw the federal labs’ contribution to collaborative, multidisciplinary R&D as potentially helpful, articulating federal labs’ promise within the context of an ‘open innovation’ system several years before the term took root [109]. While industry prized the cooperative activities above licensing or IP, the absence of a CRADA metric in the reporting statute is an early example of how empirically derived information may not appreciably affect policy in this realm [54, 110]. Our model suggests that industry input has been proven correct, in that there is a strong association between CRADAs and classical measures of success.

For example, if industry does indeed prize open innovation arrangements with federal labs, it would follow that those agencies that practice more open innovation should have more opportunity to be involved in the development of commercially-relevant licensable IP. Specifically, those agencies that engage in more CRADAs would be expected to develop more licensable IP, including patents, resulting in a higher transfer rate and licensing success rate [29, 30], not to mention licensing income. Broadly speaking, our empirical results suggest that this is indeed the case. If we normalize traditional CRADAs to the R&D budget, agencies can be ranked by normalized cost/CRADA (Table 6). Agencies with a lower cost/CRADA have a higher open innovation or ‘R&D Collaboration’ rank. Three of the top agencies (EPA, USDA, and DOE) are also among the top agencies receiving licensing income normalized to R&D budget, and three (VA, DOC, and EPA) are top performers for transfer rate.

It might be expected that agencies engaged in open innovation would be exposed to more market demand for those same technologies. For example, the VA has engaged in the largest number of CRADAs as normalized to its R&D budget over the 18-year period, yet it had low-mid range normalized license royalties. One explanation can be found in the CRADA statute itself, which provides a notable carrot for industry partners:

*“The laboratory shall ensure*, *through such agreement*, *that the collaborating party has the option to choose an exclusive license for a pre-negotiated field of use for any such invention under the agreement or*, *if there is more than one collaborating party*, *that the collaborating parties are offered the option to hold licensing rights that collectively encompass the rights that would be held under such an exclusive license by one party*.*”* (See 15 USC 3710a(b)(1))

This required provision essentially removes the risk of market competition for inventions developed under a CRADA. While those agencies that execute more CRADAs may have higher performance metrics such as higher transfer rates and higher licensing success rates, the actual licensing income is not significantly affected. The association between a higher number of traditional CRADAs and a higher portfolio exclusivity rate corroborates this explanation. Specifically, for every 10-fold increase in traditional CRADAs, there appears to be an 8% increase in exclusivity (see Table 4). Those are likely to be inventions developed under a CRADA and subject to the above provision.

### The special case of the DOD

The DOD alone accounts for over half the government’s R&D funding. At the other end of the spectrum, we have the EPA and the VA (see Table 3). Indeed, given that both agencies focus predominately on applied R&D and exhibit a relatively unrestricted approach to filing patent applications (see filing ratios in Table 3), the EPA may be the most appropriate comparator to the DOD. The model explored here treats those agencies the same, at least in most aspects. For example, transfer rate, licensing success rate, and filing ratio are within-agency measures, which can be compared between agencies. In essentially all cases and every measure, the EPA outperforms the DOD, and by far (see Table 6). One reason for this may be that, while the DOD reports as a single agency, according to the statute that defines a federal agency (See: 15 USC 3703), it actually comprises at least four distinct ‘agencies’ under the law:

*“‘Federal agency’ means any executive agency as defined in section 105 of title 5 and the military departments as defined in section 102 of such title*, *as well as any agency of the legislative branch of the Federal Government*.*”*

The above definition explicitly carves out each of the three named ‘military departments’ (i.e., Army, Navy, Air Force) as their own respective agencies, in addition to the rest of the DOD, precluding the adoption of a single set of common DOD technology transfer policies and practices. However, according to data plots showing the relative contribution of various DOD components, the Army accounts for the majority of DOD’s technology transfer transactions and does so with an R&D budget less than its counterparts [111]. If the Army were to report alone, it could well be among the top agencies in Table 6. Still, the DOD Defense Laboratories Office collates and delivers a single annual report of what statutorily should be four distinct agency accounts while each military department can point to the statute to substantiate their differences. The differences naturally contribute to frustrating delays in industry-DOD collaborations involving more than one DOD ‘agency’ [112, 113]. Amending the statute to remove the underlined part above would enable harmonized policies and practices broadly throughout the DOD.

### For good measure

Agency technology transfer reports containing prescribed measures have been required since the TTCA of 2000. One of those is “*the time elapsed from the date which the license was requested by licensee in writing to the date the license was executed”* but process metrics such as this are rarely officially reported or publicly disclosed. The concern with lengthy processes is well documented and continues to throttle effective federal technology transfer [31, 114–122]. Recognizing this, President Obama’s 2011 memorandum called to “streamline” processes and increase the “pace” of activities [123]. There are few, if any, agency- and laboratory-level reports analyzing the actual processes, and practical time and personnel costs associated with establishing technology transfer mechanisms are broadly lacking [124]. However, one study did evaluate the effects of collaboration-specific factors on inter-organizational transactional efficiency [125]. Given the overall size and continued growth of government investment in intramural R&D, more empirical studies are needed. With this in mind, here we used normalized output metrics as approximations to assess performance and investigated the associations that a host of variables have with performance measures.

We find that the average transfer rate over the past 5 reported years, approximately 40%, is exactly the same that has been calculated for university counterparts. In a similar manner, the government-wide licensing success rate of 18% and its filing ratio of 55% are also comparable to university counterparts (see Table 2). However, the extent of differences between agencies is striking (see Tables 3 and 6), raising the question, ‘What drives success?’ Through our analyses, we find a strong association between both non-patent IP policies and CRADAs and improved measures of technology transfer success. Based on the performance metrics of Table 6, the top agencies across the full 18-year period are, in order: DOC, HHS, VA, EPA, and DOE. In the most recent 5-year period, the top performers in order are: EPA, HHS, DOC, VA & DOE (tied). For the other agencies, implementing and taking the following actions may be associated with improved technology transfer performance:

1. DOI: adopt non-patent IP policies and a more discerning approach to patent filing
2. USDA: to the extent possible, adopt a copyright policy and employ a more discerning approach to patent filing
3. DOD: apply a more discerning approach to patent filing; consider adopting a copyright policy minimally for FFRDCs and other contractor-operated labs
4. DOT: adopt non-patent IP policies to the extent possible and continue to apply rigor to disclosure review
5. NASA: adopt a material transfer policy

In summary, while most prior performance studies focus on patents and licenses, our analysis uncovers the importance of R&D collaboration and non-patent IP policies to technology transfer success. Specifically, we find agencies that employ an open and comprehensive approach to innovation management have measurably higher successes in traditional technology transfer metrics. We find a significant association between a higher number of traditional CRADAs and higher transfer and licensing success rates. Non-patent IP policies enabled through long-standing agency-specific authorities or a more inclusive interpretation of pan-government statutes are similarly associated with significant improvements in performance as measured by the transfer and licensing success rates, as well as with a more prudent approach to invention review and patent filing. Our research underscores the importance of evaluating how the utilization of external R&D collaboration and coordination of non-patent IP policies affects the supply-side and demand-side of federal technology transfer performance.

### Generalizing key findings

As we introduced at the outset of this study, national government leaders and senior policymakers around the world–from Beijing to Tel Aviv to Berlin to Washington, D.C.–are all increasingly investing in and leveraging public sector R&D entities and activities to recover from unexpected external shocks and rebuild core components of their respective infrastructures and economies. These clear official mandates and policy directives emphasize the paramount importance of innovation as a chief driver of future growth, greater prosperity, and stronger security. In this context, we believe that our study of U.S. federal agencies’ technology transfer activities and outcomes offers initial empirical evidence and actionable insights that may be more broadly applicable to overcoming similar challenges with managing government-industry innovation processes in other countries as well.

For example, ongoing government efforts to prioritize national labs as strategic resources, coupled with incentives for external R&D collaboration, and mechanisms for sharing non-patented IP are all essential parts of the available managerial toolkit for supporting technology transfer. We find evidence that U.S. federal agencies with a greater utilization of FFRDCs have lower agency-driven technology transfer performance in terms of supply-side metrics. On the demand-side, we find that greater FFRDC utilization is associated with greater licensing income and lower portfolio exclusivity. We now turn to explaining how these findings may potentially inform comparable efforts in other countries.

Recall that China is in the midst of dramatically expanding its system of national labs from 200 to 700 institutes administered by the Chinese Academy of Sciences. As noted earlier, France is already allocating 80% of its public R&D spending to the 10 institutes of the CNRS, while its European counterpart, Germany, is deploying a massive technology-focused economic stimulus package delivered through three distinct federal- and state-sponsored R&D networks consisting of the Helmholtz Association of German Research Centers, the Max Planck Institutes, and the Fraunhofer Institutes. To facilitate stronger technology transfer from national labs, Israel is revising its policy in the governmental R&D sector. Other countries such as India, Turkey, Uruguay, and South Africa are putting greater emphasis on developing national innovation systems to pursue scientific breakthroughs that may yield long-term economic growth [7–9, 126–128].

Our findings suggest that simply increasing the overall portion of public R&D funding allocated to these national labs may not necessarily produce corresponding increases in supply-side metrics. In fact, our findings spanning several years of U.S. federal agencies suggest that greater overall utilization of national labs may actually produce lower supply-side performance measured in terms of *filing ratio*, *transfer rate*, and *licensing success rate*. There may also be a considerable time lag between the initial investment and the observable inventive outputs. The knowledge generated as a public good through R&D activities undertaken by national labs is cumulative and highly interdependent in terms of drawing from multiple disciplines and domains. This further suggests that the pathways to commercialization may require a longer-term and sustained initiative that includes a range of external R&D collaborators in the private sector and new types of arrangements for handling non-patented IP.

Our findings also indicate that the trade-off between the demand-side metrics of *licensing income* and *portfolio exclusivity* must be carefully managed. On the one hand, if a government intends to help establish a technical standard based on essential IP, it may actively promote licensing arrangements with a full spectrum of industry participants. In such cases, lower levels of licensing income do not necessarily signal a lack of commercial viability. In fact, if the licensing rights are offered at nominal fees, the associated income might indicate exactly the opposite, namely that widespread adoption of the standard is occurring. This is why the interpretation of the demand-side metric of licensing income should always be evaluated in conjunction with the level of portfolio exclusivity. On the other hand, if a government intends to nurture a particular sector, it may offer exclusive licensing terms with only a select few industry participants. Here, the policy aims may be completely different than promoting standards, and the approach is to provide essential IP as a potential source of competitive advantage to protect certain actors within a promising industry sector. Again, the demand-side metrics of licensing income and portfolio exclusivity must be evaluated and interpreted in the context of the overarching policy objectives and regulatory frameworks of the respective countries that are implementing these metrics.

As a further example that the key findings of this study may be generalized, consider that by allocating and aligning annual R&D budgets with their missions, federal technology managers are able to ascertain the utilization of FFRDCs for their respective agencies. Rather than using CRADAs or traditional contracts, greater emphasis on FFRDCs utilization for managing R&D might potentially reduce supply-side while amplifying demand-side technology performance. By decentralizing core knowledge generation and shifting it from agency-operated sites to contractor-operated FFRDCs, this change may advance the technologies closer to commercialization and increase demand-side performance. Recalling our previous description of the role of national labs in China, France, and Germany, our findings suggest that the extent to which the day-to-day operations of these labs are administered by internal government agencies or external private contractors may have a meaningful impact on the observed levels of supply-side and demand-side metrics for technology transfer performance.

In addition, our findings regarding the importance of CRADAs and non-patent IP policies as predictors of success align with reports investigating university-industry collaborations from many regions. Companies operating in the same sector can benefit from knowledge spillovers within a geographic location, and university-industry collaborations can solidify such geographic industrial clusters [129]. CRADAs are inherently collaborative, and collaborations based on clear shared goals and honest, open dialogue enhance university-firm performance [130, 131]. Evidence from comparative case studies reported in the literature and our quantitative analyses in this study suggests that the same is likely true for government-firm collaborations [55, 132, 133]. Similarly, the strategic significance of non-patent IP is increasingly being recognized [134, 135], even as the approach to knowledge management and appropriation of non-patent IP requires more nuance than the management of patent IP [136, 137].

For example, proposals based on expectations that blockchain technology may facilitate a practical approach to knowledge management of these forms of IP, and thereby enhance their commercialization potential, are being developed and disseminated [138, 139]. In another example that is especially pertinent to the outbreak and aftermath of the coronavirus, there is renewed interest among national governments and transnational actors such as the World Health Organization to facilitate greater pooling of cohort data from clinical trials, foster more open sharing of biological materials, and encourage the voluntary waiving of certain IP rights to accelerate medical R&D for combating COVID-19 [140–142]. Hence, new forms of CRADAs and MTAs may emerge in the near future, which only underscores the importance of identifying valid and meaningful ways to measure technology transfer activities and performance over time.

### Limitations and future directions

Our study is limited in ways that further research may overcome in the future. Based on publicly available databases managed by the U.S. federal government, our sample timeframe was restricted from 1999–2016 and sample size to 127. However, due to this limitation, we carefully interpreted the results and determined the effect sizes. Future research may be able to utilize a larger sample when federal agency data is made publicly available and provides the opportunity to extend our initial findings.

Our empirical findings provide guidance for formulating evidence-based policy reforms that are tailored to the mandated missions and statutory constraints of each agency. We believe that our findings could be the starting point for new streams of research on this topic that may be generalized and applied to other countries and contexts. We encourage future researchers to continue to investigate this area and help provide policymakers with essential tools that may drive a policy consensus regarding best practices for improving technology transfer performance within and across government agencies around the world.

As other countries such as China, in particular, continue to rapidly expand their own systems of national labs and make more managerial data available for external use by interested parties, it may be feasible to conduct comparative quantitative analyses of technology transfer across multiple countries during the same timeframe. In addition, there may be opportunities to expand our work by examining the extent to which multiple networks of public sector R&D institutes in countries such as Germany and Israel are complementary or competitive with each other in creating and commercializing scientific and technical breakthroughs. The more decentralized and distributed approach to public sector R&D followed by Germany may provide an interesting contrast to the more centralized and concentrated approach followed by its fellow European Union member, France. We believe that our study highlights a promising avenue for future research and scholarly inquiry. Although countries may differ substantially in their political systems and economic priorities, they may continue to encounter similar challenges when analyzing and formulating future policies to achieve their respective national innovation goals. Our study suggests that understanding the most effective practices and process for managing government-industry technology transfer on the supply-side as well as the demand-side is a matter of strategic importance that may help leaders and stakeholders in the public and private sectors make progress towards building a policy consensus and reaching their development objectives.
